# Prediction of intrinsic and extraneous cognitive load with oculometric and biometric indicators

**DOI:** 10.1038/s41598-025-89336-y

**Published:** 2025-02-12

**Authors:** Merve Ekin, Krzysztof Krejtz, Carlos Duarte, Andrew T. Duchowski, Izabela Krejtz

**Affiliations:** 1 Institute of Psychology, SWPS University, Warsaw, 03-815 Poland; 2 LASIGE, Faculty of Sciences, University of Lisbon, Lisbon, 1649-004 Portugal; 3 School of Computing, Clemson University, Clemson, SC 29631 USA

**Keywords:** Intrinsic cognitive load, Extraneous cognitive load, Eye tracking, Bio-metric, Linear discriminant analysis, Psychology, Cognitive neuroscience

## Abstract

This study focused on the prediction of intrinsic and extraneous cognitive load using eye-tracking metrics, heart rate variability, and galvanic skin response. Intrinsic cognitive load is associated with the inherent complexity of the mental task, whereas extraneous cognitive load is related to the distracting and unrelated elements in the task. Thirty-three participants (aged $21.24 \pm 3.51$) performed different levels of mental calculations to induce intrinsic cognitive load in the first task and a visual search task to manipulate extraneous cognitive load in the second task. During both tasks, participants’ eye movements, heart rate, and galvanic skin response were continuously recorded. Participants’ working memory was controlled. Subjective cognitive load was also assessed following each experimental task. A discriminant model, consisting of oculo- and bio-metric indicators, could discriminate between cognitive loads (intrinsic vs.extraneous) and levels (low vs.high). In particular, average fixation duration, average saccade amplitude, and $\mathscr {K}$ coefficient each have an impact on the model. In addition, task difficulty may be distinguished by the Low-High Index of Pupillary Activity (LHIPA) and heart rate variability.

## Introduction

Cognitive Load Theory (CLT) models the limited working memory capacity in tasks requiring cognitive processing^1^. CLT and previous research have distinguished three additive types of cognitive load: Intrinsic Cognitive Load (ICL), Extraneous Cognitive Load (ECL), and Germane Cognitive Load (GCL)^2^. ICL is related to the difficulty and complexity of the task, referred often as the mental effort needed for a task completion^1, 3^. ECL is the interaction between elements manipulated by external or redundant factors^1, 3^, and depends on the stimuli presentation format, spatial and temporal organization, or visual difficulty^4^.

The main difference between ICL and ECL is the source of mental effort. ICL emphasizes the nature of the material and mental load resulting from cognitive processing e.g., reasoning or learning, while ECL emphasizes the effort spent on unrelated elements in the learning environment that are not due to the complexity of the task^2, 4^ but visual design of the stimuli. DeLeeuw et al.^5^ compared types of cognitive load in a multimedia learning environment and reported that ECL and ICL could be distinguished by manipulating redundancy and complexity, respectively. In addition, GCL is related to the construction of new resources and schemes indicating the transfer process of new information during learning^1, 3, 5^. Duchowski et al.^6^ demonstrated that cognitive load could be manipulated with the complexity of abstract visual stimuli while performing a visual search task. That manipulation can be related to Wolfe’s Guided Search Model^7^ which postulates that the salience of the target relative to distractors influences visual search. The model proposes that when distractors are similar to the target (in terms of features like shape or orientation, e.g. angular slope), the visual search process is more effortful, engaging more bottom-down processes. In other words, the less salience difference between the target and distractors, the more extraneous CL.

Cognitive Load (CL) can be assessed using a variety of methods, including subjective methods such as self-reporting, and physiological methods using sensor technologies such as eye tracking (ET), Galvanic Skin Response (GSR), and Heart Rate Variability (HRV)^8, 9^. Eye tracking is a non-invasive and cost-effective method used for measuring gaze patterns and gaze positions, which can be informative for cognitive load^10^. Bio-signals are also used to detect emotions and cognitive load and provide objective information and unbiased data^11–13^. As cognitive load is affected by internal and external factors such as cognitive ability, prior individual experience, the number of tasks, and physical conditions^11, 14^ assessing signals from various parts of the body such as the skin, eyes, or heart can contribute to the validity and reliability of CL prediction^14, 15^. Studies investigating the prediction of general cognitive load-based on physiological data in tasks requiring mental assessment have found classification accuracy to be quite high^8, 16^. Rahman et al.^17^ used a Machine Learning (ML) classification model of cognitive load based on fixation and saccade parameters from facial image sequences and achieved the best performance in differentiating CL for the support vector machine and linear kernel function compared to other ML algorithms and kernels. In addition, Skaramagkas et al.^18^ reported that eye metrics were useful features to classify high and low general CL with multi-class classification tools.

Zu et al.^4, 10^ were one of the first to demonstrate, based on complexity, redundancy, and transfer performance, that eye-movement characteristics may discriminate between three different types of cognitive load in learning scenarios. Using hierarchical regression analyses, they showed that average fixation duration was most affected by extraneous load, mean saccade peak velocity was most affected by germane load, and transitions between text and animation in the learning material were most affected by intrinsic cognitive load (the more transitions the higher the load).^10^ Pupil diameter change was sensitive to both extraneous and germane load^10^. These results, although promising, might also be seen as very specific to the task used in the experiments with limited generalization power thus needing further empirical investigation.

A recent study by Li et al.^19^ indicated that physiological data from touch gestures and eye tracking might be an effective method for real-time CL prediction. The machine learning approach achieved 0.94 accuracy using touch gestural and eye-movement characteristics. They demonstrated that pupil diameter, fixations, and saccades have satisfactory power to predict cognitive load in learning (see also Zagermann et al.^20^). Li et al.^19^ suggested that pupil diameter and total fixation time increase with the increase in load while saccade amplitude decreases with cognitive load. They did not distinguish between different types of cognitive load, however.

A brief survey of existing literature shows that there is no consensus on fixation- and saccade-related metrics for assessing cognitive load in various cognitive processes. Rivecourt et al.^21^ examined how eye metrics change during flight simulator training and found a decrease in fixation duration with increasing task demand. Mallick et al.^22^ used a video game to increase cognitive load and reported a decrease in fixation duration and an increase in saccadic amplitude. In addition, Krejtz et al.^23^ derived the $\mathscr {K}$ coefficient, calculated using fixation duration and subsequent saccade amplitude, as a dynamic index between ambient and focal visual attention, which are two general modes of the visual attention process (see also Velichkovsky et al. (2005)^24^). Longer fixations and shorter saccades characterize focal attention, observed during increased perceived difficulty and cognitive load^23, 25, 26^. A study investigating visual search tasks with different levels of difficulty also indicated that a higher number of fixations was related to increased cognitive load^27^. On the other hand, Liu et al.^28^ showed that high cognitive load leads to decreases in the number of fixations and longer fixation duration. In a study by Walter and Bex^29^, as the cognitive load in a visual search task increased with task difficulty, fixation duration increased. There are other studies showing that as task complexity increases, fixation duration increases^30, 31^, while saccadic amplitude decreases^32^. In conclusion, although there is no clear consensus, fixation duration and saccade amplitude seem to be useful metrics for measuring cognitive load. As task complexity and cognitive demands increase, fixation duration generally increases and saccade amplitude generally decreases.

Another important eye-related metric is pupil dilation, which is an involuntary reflex used to assess cognitive load^33^. Hess and Polt^34^ suggested that cognitive load could be measured by changes in pupil diameter. They demonstrated that pupil responses reflect mental activity and vary with task difficulty. Studies have shown that pupil diameter tends to increase with increasing cognitive load, which is known as the task-evoked pupillary response^35, 36^. Batmaz et al.^37^ who investigated the pupillary response during a performance-based task, found that the pupil dilated with increased cognitive load. In addition, studies have reported that pupil dilation increases in difficult mental arithmetic tasks compared to easy tasks^38, 39^. There are also special metrics to discriminate cognitive load using pupil diameter, e.g. the Low-High Index of Pupillary Activity (LHIPA). LHIPA represents the changes between low- and high-frequency pupil diameter oscillations to predict cognitive load^40^. Duchowski et al.^40^ reported a negative relationship between both task difficulty and perceived difficulty of mental tasks (e.g., *n-back*) and LHIPA.

Cognitive load can also be assessed using biometrics, such as HRV and GSR, which are altered by physiological arousal under stressful conditions, such as mental tasks^41^. Heart rate is the number of heartbeats per minute. Heart rate variability is the variation in the time interval (ms) between successive heartbeats, the Inter Beat Intervals (IBIs)^42, 43^. HRV is an index of autonomic control of the heart^44^. HRV is controlled by the sympathetic and parasympathetic nervous systems (SNS-PNS); therefore, high HRV occurs with high PNS and better coping with stress under stressful conditions^45, 46^. Previous research has shown that better stress management during cognitive tasks increases both cognitive performance and HRV, with an increase in parasympathetic nervous system activation^47–49^. Therefore, a negative correlation between cognitive load and HRV during mental tasks is likely, despite the scarcity of studies^8, 50, 51^. However, Solhjoo et al.^52^ showed a high positive correlation between perceived intrinsic cognitive load and HRV frequency. The effect of HRV reflects the baseline autonomic function, whereas active HRV captures the real-time physiological responses to cognitive demands during a task. Studies have demonstrated that active HRV frequently decreases with increasing cognitive load^53^ however, passive HRV may not directly reflect task-related changes.

Galvanic Skin Response (GSR), also known as the electrodermal activity or skin conductance, provides a real-time measure of cognitive load during mental tasks and is measured by the sweat glands innervated by SNS^54^. Studies have shown that GSR increases as cognitive tasks become more difficult,^55, 56^ and GSR can discriminate cognitive load based on difficulty levels using classification algorithms^54^. However, Ikehara et al.^57^ found a decrease in GSR with increasing task difficulty when the task is too simple. There is also research that has found no relationship between GSR and CL^16^. Existing literature has shown that physiological measures are effective in understanding cognitive load, but there is still much to be done in this area^8, 16^.

In this study, we used eye-tracking measures, heart-rate variability, and skin conductance to discriminate between intrinsic cognitive load (ICL) and extraneous cognitive load (ECL) induced by mental calculations and visual search tasks, respectively. To the best of our knowledge, this is the first study to use two different types of eye-tracking measures: traditional first-order metrics (e.g., average fixation duration, average saccadic amplitude, pupil size) and second-order metrics (e.g., $\mathscr {K}$ coefficient of ambient-focal attention, Low-High Index of Pupillary Activity) to differentiate between intrinsic and extraneous cognitive load. The contribution of this study is twofold. First, we present the use of the second-order metrics (ambient-focal attention and LHIPA) to discriminate between cognitive load types (ICL vs ECL), and task difficulty (low vs high). Second, we explore the combination of other biometrics (GSR and HRV) with eye-tracking metrics to more accurately capture the physiological response to task difficulty and cognitive load type. Overall, the study aimed to differentiate between intrinsic and extraneous cognitive load using physiological measures to develop a predictive machine-learning model. The main hypothesis of this study is related to the discriminatory model of low vs high intrinsic and extraneous cognitive load with a combination of physiological measures.

## Method

The present study was a $2\times 2$ within-subjects design experiment with two independent factors, cognitive load level (high vs. low) and cognitive load type (intrinsic vs. extraneous). The cognitive load type was manipulated with the task type (mental calculations vs. visual search, respectively). The cognitive load level was manipulated with the tasks’ difficulty (high vs. low).

### Participants

This study initially consisted of 34 English-speaking students of social sciences. Participants volunteered in the study in exchange for the credit points required by their study programs. One participant was excluded from the study due to problems with eye-tracking recording, and the final number of participants was 33 (24F, aged $21.24 \pm 3.51$). The dominant hand of the participants, except for one participant, was the right hand. Before the experiment, all participants were informed about the study and the study method. They signed an informed consent form, which included details of the study and participant’s rights. No personal data were collected during the recording. The study and all methods were performed in accordance with the relevant guidelines and regulations of the Declaration of Helsinki. The study was approved by the Ethical Review Board at the Faculty of Psychology, SWPS University (decision no: 47/2023).

### Intrinsic and extraneous cognitive load tasks

**Intrinsic Cognitive Load:** The Mental Calculation Task was inspired by the procedure used in previous research on cognitive load^58, 59^. In this task, participants were asked to complete 24 trials of mental calculations, consisting of six trials of each condition: short-easy, short-hard, long-easy, and long-hard. The short-easy trials contained a one-digit number and a two-digit number, there were no borrowing and carrying steps necessary during addition or subtraction. The short-hard trials also contained a one-digit number and a two-digit number. However, these calculations required borrowing and carrying steps in the calculations. The long-easy trials contained two two-digit numbers, and no borrowing and carrying steps were needed during addition or subtraction. The long-hard calculations contained two two-digit numbers and required borrowing and carrying steps during addition or subtraction (e.g., short-easy ($26\!-\!2\!=\!24$), short-hard ($38\!+\!7\!=\!45$), long-easy ($32\!+\!24\!=\!56$), long-hard ($64\!-\!27\!=\!37$)). There were three addition and three subtraction operations for each condition, and all numbers were randomly selected based on the predetermined conditions of difficulty.

**Fig. 1:**
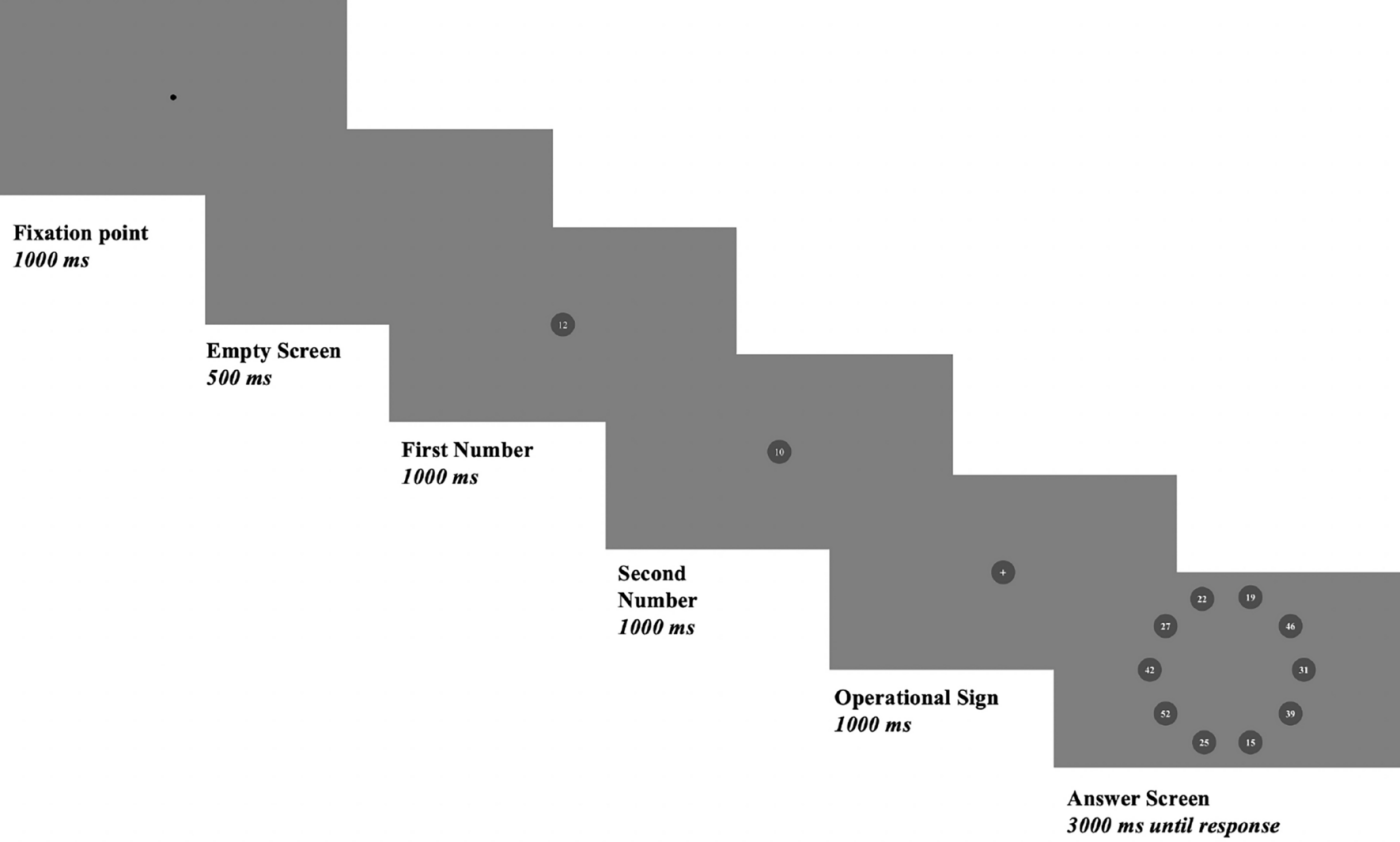
The schematic presentation of a trial in the Mental Calculation Task. Note: Physiological measures were recorded during the answer screen presentation.

Figure 1 presents the flow of an exemplary trial in the MCT. Each participant in each trial was presented with a fixation screen for 1000 ms, a blank screen for 500 ms, the first number for 1000 ms, the second number for 1000 ms, the operator for 1000 ms, and the answer screen for a maximum of 3000 ms to respond. Thus, each trial lasted approximately 7500 ms. Physiological measures were recorded during the answer screen. Both screens used a gray background with circular points. In both tasks, participants had to identify the “target” on the screen by moving their eyes to find the target/correct response, so they exhibited fixations and saccades necessary to respond.

Participants received the following instruction: “In this task, you will see two numbers and an operator in a row. Your task is to calculate the operation and to click with the mouse at the correct answer as quickly and correctly as possible”. The operator and numbers were displayed in a black circle ($\#\ 4C4C4C$) subtending $0.69 \times 0.69$ degrees visual angle ($width \times height$) on a gray background ($\#\ 808080$) in the center of the screen. Participants were then shown a response screen containing ten possible answers, and the task was to click on the correct answer. A fixation point was displayed in the center of the screen between each trial for one second. The fixation points between trials were added as an “overlap” condition, serving to prepare participants for the next trial. However, no instructions were provided to direct their attention to the fixation point. Before the main trials, participants were given 4 training trials (one for each condition).

**Fig. 2:**
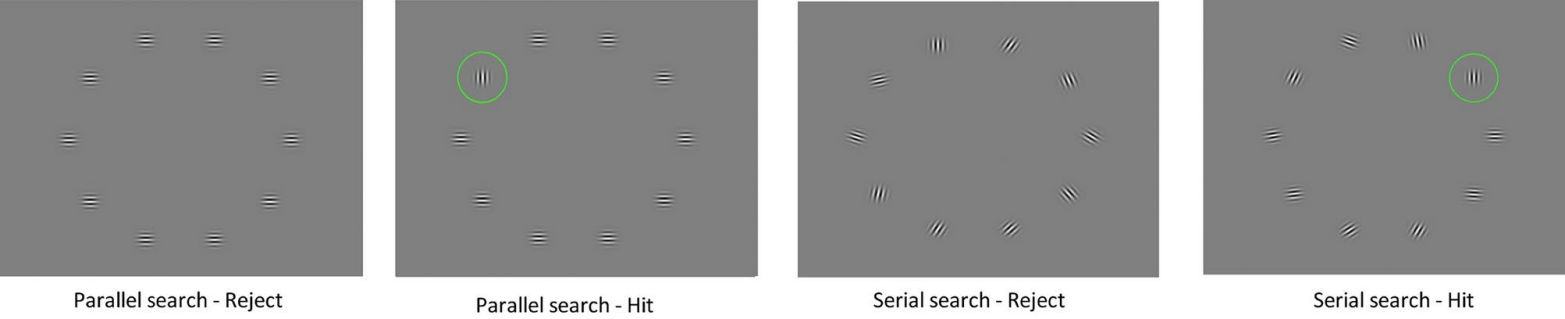
Visual stimuli of the Visual Search Task. Note: The green circle indicates the target for “hit” condition and is representative. Physiological measures were recorded during each stimulus presentation.

**Extraneous Cognitive Load:** The Visual Search Task (VST) was inspired by the task described by Krejtz et al.^23^. The participant’s task was to find the visual target, which was the vertically oriented Gabor patch, among ten circularly presented Gabor patches^23, 60^. All patches were $\hbox {2}^{\circ }$ in size and were located $\hbox {8}^{\circ }$ from the center. There were two visual search types, namely parallel and serial search. While the former consisted of ten horizontally oriented Gabor patches, the latter consisted of ten randomly oriented Gabor patches varying between $\hbox {20}^{\circ }$ and $\hbox {340}^{\circ }$. In addition, there were two target presence conditions, namely hit and reject conditions. While the target was presented in the hit condition, it was absent in the reject condition (Fig. 2). Physiological measurements were recorded during the screens presenting the stimuli (see Fig. 2). The duration of each trial was determined by the response time of the participants, as there was no time limit for providing answers.

The instruction of the VST was as follows: “In this task, we will show you Gabor patches. Your task is to find a Gabor patch oriented vertically. If you think that the vertical patch is present, you should press M. If you think that the vertical patch is absent, you should press B”. In the procedure, participants were first presented with training including a total of eight trials (four parallel searches: two hits, two rejects, and two serial searches: 2 hits, 2 rejects). Participants received feedback, “Correct!” or “Wrong!” based on their responses during the training. The main task consisted of a total of 20 trials for two visual search types (10 parallel and 10 serial searches), and there was no feedback. The visual search types were presented in a random order in the training and the main task. For serial and parallel searches, there were two target present conditions, hit and reject which were randomised, leading to the various numbers of hits per search type (from 2 to 5).

For both tasks, we analyzed data from the response time window. For the mental calculation task, it was the time window for providing the result of the mental calculation, the answer screen presented up to 3000 ms or until a response (see Fig. 1). For the visual search task, we also analyzed data recorded over the answer screens which were the same as stimuli presentation screens (see Fig. 2).

### Apparatus and research materials

The study used the Gazepoint GP3-HD eye tracker to measure gaze data. The Gazepoint Biometrics / Finger Sensor Module recorded galvanic skin response and heart rate variability. The devices sampled at a rate of 150 Hz and were synchronized with each other so that all physiological data could be recorded at the same time. The system exhibits reliable sensitivity and accuracy in capturing eye-tracking and psychophysiological data, which makes it a cost-effective tool for assessing cognitive load and other biometric responses^61^. The monitor used for the task presentation was 21.5 inches, 59.94 refresh rate, and $1920 \times 1080$ screen resolution.

#### The visual Digit Span Task (Visual DSPAN)

The DSPAN measures working memory capacity^62, 63^. The reason for choosing the visual version of the task was that the participants were non-native English speakers^64^. They saw forward and backward digit sequences (starting from 3 digits) for one second, and recalled them by choosing the digits from a circle of digits from zero to nine with the mouse. If they gave a correct answer, the length of the sequence was increased, otherwise, the length of the sequence remained the same. After completing the 14 trials for the forward and backward conditions separately, the task was over. The task took approximately 15 minutes to complete and was administered using Inquisit 5 lab^65^. If participants recall all digits in the correct order, a sequence is scored correctly. As an indicator of working memory, we selected the backward DSPAN score. The reason for choosing the backward score is that it typically requires more working memory and cognitive manipulation, which is indicative of cognitive load^66^.

#### The NASA Task Load Index (NASA-TLX)

The NASA-TLX is a tool commonly used as a subjective measure of cognitive load^67^. The task comprised six questions related to mental, physical, and temporal demands, frustration, effort, and performance. The scale ranges from 0 to 20 (very low – very high). The duration of the task was almost one minute and it was administered using Inquisit 5 lab^65^.

### Procedure

Participants signed the consent form and were informed about the study before completing the visual DSPAN task to measure working memory capacity. Each participant then sat in front of the screen and placed their left middle and ring fingers on the finger sensor. The distance between the participant and the eye tracker was 570 mm. This was followed by a 5-point calibration and validation to map the gaze positions before each task. The MCT and VST, which were presented in random order, lasted approximately 15 and 10 minutes, respectively. The trials of MCT were presented in a randomized order for each participant. In the trials of VST, we randomised the parallel and serial search blocks, and the presentation of hit vs reject trials within each block was also randomised. Prior to the beginning of the task, all participants were presented with a baseline screen, which was a blurred image of the answer screen for MCT and the stimulus presentation screen for VST, during physiological measurements. The purpose of the baseline screen was to calculate the differences between the pupil size during the tasks and the baseline for each participant. After each task, the NASA-TLX was used to subjectively measure cognitive load. The finger sensor was cleaned with an alcohol-cleaning wipe before each participant. Ambient light was also measured for each participant recorded at 11 lux, on average. The background colour of the answer screen recorded for MCT and the stimulus screen recorded for VST was the same. The position and size of the stimuli were also the same for both tasks.

### Data processing

All data were preprocessed using Python and R computational language scripts for detecting fixations and saccades with a dispersion-based algorithm. In the preprocessing stage, outlying fixation durations and saccadic amplitudes (over $M\pm 1IQR$) were reduced to the highest non-outlying value. Intrinsic and Extraneous Cognitive Load (ICL and ECL, respectively) were analyzed using eye-tracking metrics, including fixation duration, saccade amplitude, $\mathscr {K}$ coefficient, pupil dilation, and Low-High Index of Pupillary Activity (LHIPA), and biometrics, namely galvanic skin response and heart rate variability. While the data for ICL were obtained from the mental calculation task, the data for ECL were obtained from the visual search task.

The $\mathscr {K}$ coefficient introduced by Krejtz et al.^23^ was obtained using the standardized *z*-score of fixation duration and saccade amplitude, and ambient/focal visual scanning was calculated per individual. The $\mathscr {K}$ coefficient score is measured on a scale of -1 to +1, with positive values indicating focal visual attention and negative values indicating ambient visual attention. The $\mathscr {K}$ coefficient is calculated for each participant, mean differences between *z*-score of each saccade amplitude ($a_{i+1}$) and its preceding $i^{\text {th}}$ fixation duration ($d_i$):

$$\begin{aligned} \begin{array}{cc} \mathscr {K} _i = \frac{d_i - \mu _d}{\sigma _d} - \frac{a_{i+1} - \mu _a}{\sigma _a} \quad&\quad \mathscr {K} = \frac{1}{n} \sum _{n} \mathscr {K} _i \end{array} \end{aligned}$$

where: $\mu _d$ is the mean fixation duration, $\sigma _d$ is the standard deviation of fixation durations, $\mu _a$ is the mean saccade amplitude, $\sigma _a$ is the standard deviation of saccade amplitudes, and $n$ is the number of fixations.

LHIPA is also a metric to discriminate cognitive load similar to IPA^40^. The main difference between the metrics is that LHIPA considers the ratio of low and high-frequency bands contained in the wavelet decomposition of the pupil diameter signal, *x(t)*. LHIPA is computed as the ratio of low to high frequency ${x^{1/2\log _2{(n)}}_{\psi }(t)}/{x^{1}_{\psi }(2^{1/2\log _2{(n)}}t)}$ of the wavelet coefficients $\psi _{j,k}(t)$ of the pupil diameter signal $x(t)\!=\!\sum _{j,k = -\infty }^{\infty }{c_{j,k}\psi _{j,k}(t)},$ $j,k\!\in \!\textbf{Z}$, and is expected to decrease with increased cognitive effort. In addition, pupil dilation was calculated from the inter-trial change^38^ based on the differences between pupil diameter in the mental task and the baseline display presented before the mental tasks. The baseline screen allows individual changes in pupil size to be determined for each participant, independent of cognitive load.

GSR evaluates skin resistance (in Ohms), and typically ranges between $10k\Omega$ and $2M\Omega$, and the HRV was calculated using a moving average over three beats to reduce the noise due to possible finger movements. In the preprocessing stage, the standard deviation of RR intervals was calculated in order to HRV. It is a standard time-domain HRV measure^42, 68^ which provides continuous and objective indicators of cognitive engagement. We used the active mode of the HRV recording (during active task performance)^43, 69^. The Inter-Beat Intervals (IBI) values were calculated using the R script based on Matlab script provided by GazePoint Inc. In the raw stream of HRP - a “heart rate pulse signal is unitless but proportional to an ECG signal”^70^. In the HRP signal local peaks were found with the findpeaks function from “gsignal” R package for signal processing^71^. The distance between peaks constituted Inter Beat Intervals values, “known as the beat to beat interval or RR interval”^70^. The Heart Rate Variability (HRV) values were calculated as standard deviations of IBI values for each trial and each participant in the study.

### Statistical analysis plan

Linear Discriminant Analysis (LDA), a supervised machine learning approach for separating multi-class groups, was performed with MASS package^72^ in R, the language for statistical computing^73^. We investigated the sensitivity of oculo- and biometrics, controlling for working memory in discriminating between intrinsic and extraneous cognitive load and their low and high levels. First, for the LDA, we created factor variables from the two tasks with four conditions, indicating low and high levels of intrinsic and extraneous cognitive load. The low level of ICL was presented by short calculations (”short-easy” and ”short-hard” of the mental calculation task), whereas the high level of ICL was presented by long calculations (”long-easy” and ”long-hard” of the mental calculation task). The low level of ECL was presented by parallel calculations (”parallel-hit” and ”parallel-reject” of the visual search task), whereas the high level of ECL was presented by serial calculations (”serial-hit” and ”serial-reject” of the visual search task).

Second, in the LDA model, we used predictors of three groups: a) the first-order oculometrics used in previous research to discern cognitive load (average fixation duration, average saccade amplitude, and pupil dilation), b) the second-order oculometrics ($\mathscr {K}$ coefficient and LHIPA), and c) biomarkers (HRV and GSR). Additionally, working memory capacity (the DSPAN backward score) was also included as a predictor in our LDA model. The model was performed on the transformed training dataset of $80\%$, randomly chosen cases from the entire original dataset. The LDA model was evaluated for classification accuracy using AUC - ROC curve, confusion matrix, precision, recall, and F1 score. The accuracy of the LDA model is presented with a confusion matrix, accuracy rates and ROC curve analysis to assess classification performance. Before presenting the main model, which simultaneously distinguishes between types and levels of cognitive load, we ran two separate LDA models to check the accuracy of the first model for distinguishing between types of cognitive load, and the second model for distinguishing between levels of cognitive load. In brief, the preliminary models evaluate two conditions (one for cognitive load levels, and another for cognitive load types), whereas the main model assesses both cognitive load levels and types. We report all statistics for the main model and only the classification accuracy for the separate models.

## Results

### Accuracy rates and behavioral metrics

The ANalysis Of VAriance (ANOVA) for accuracy in the Visual Search Task indicated a significant main effect for the visual search types (*F*(1,33) = 33.10, *p* < 0.001). The parallel search (*M* = 0.91, *SD* = 0.03) was observed to be more accurate than the serial search (*M* = 0.68, *SD* = 0.03). The ANOVA was also performed for accuracy in the Mental Calculation Task, which showed two significant main effects, for difficulty level (*F*(1,33) = 11.50, *p* < *0.001*) and the number of digits (*F*(1,33) = 18.86, *p* < 0.001). The main effect of difficulty level indicated that the easy condition (*M* = 0.93, *SD* = 0.01) was more accurate than the hard condition (*M* = 0.89, *SD* = 0.02). The level of accuracy was higher for the short digits (*M* = 0.97, *SD* = 0.01) than for the long digits (*M* = 0.88, *SD* = 0.02). The interaction effect between difficulty levels and number of digits was also significant (*F*(1,33) = 5.78, *p* = 0.02). In terms of accuracy, the long-hard condition had the lowest value, while the short-easy demonstrated the highest accuracy. The means and standard deviations of the interaction effect for each difficulty level in both tasks are presented in Table 1.

**Table 1:** Means and standard deviation of oculo- and bio-metrics in experimental conditions.

	Fixation Duration ms)	Saccade Amplitude (px)	$\mathscr {K}$ coefficient	HRV (sec.)	GSR (ohms)	LHIPA	Pupil Dilation	Accuracy
Mental Calculation Task								
Short - Easy	$366.01\pm 126.30$	$240.47\pm 97.95$	$0.35\pm 0.55$	$0.24\pm 0.36$	$354849.01\pm 279677.79$	$1.40\pm 3.59$	$1.52\pm 10.87$	$0.98 \pm 0.01$
Short - Hard	$440.38 \pm 153.63$	$285.59 \pm 95.16$	$0.38 \pm 0.61$	$0.16 \pm 0.25$	$354763.99 \pm 282342.00$	$4.02 \pm 4.93$	$2.80 \pm 11.24$	$0.97 \pm 0.01$
Long - Easy	$425.03 \pm 156.40$	$300.56 \pm 98.54$	$0.28 \pm 0.64$	$0.17 \pm 0.28$	$348591.14 \pm 271746.00$	$4.71\pm 5.02$	$3.30 \pm 12.25$	$0.94\pm 0.02$
Long - Hard	$428.68 \pm 141.70$	$325.11 \pm 97.02$	$0.19 \pm 0.60$	$0.12 \pm 0.21$	$346919.31 \pm 271564.31$	$6.45 \pm 4.48$	$4.72 \pm 11.99$	$0.82 \pm 0.04$
Visual Search Task								
Parallel - Hit	$406.53\pm 153.45$	$248.82\pm 106.19$	$0.30\pm 0.61$	$0.01\pm 0.03$	$603613.61\pm 2211719.30$	$0.85\pm 3.07$	$-3.59\pm 8.20$	$0.91\pm 0.04$
Parallel - Reject	$451.53\pm 175.54$	$325.99\pm 73.89$	$0.12\pm 0.58$	$0.01\pm 0.02$	$1157919.87\pm 3738333.59$	$4.09\pm 5.65$	$-3.11\pm 5.63$	$0.89\pm 0.04$
Serial- Hit	$545.50\pm 242.07$	$283.69\pm 100.79$	$0.51\pm 0.81$	$0.01\pm 0.02$	$892198.06\pm 2776476.84$	$4.41\pm 5.69$	$-2.26\pm 6.03$	$0.67\pm 0.05$
Serial - Reject	$529.24\pm 195.96$	$304.69 \pm 92.80$	$0.39\pm 0.69$	$0.01\pm 0.02$	$609290.43\pm 1960143.51$	$6.78\pm 5.83$	$-3.19\pm 7.87$	$0.67\pm 0.04$

In this study, NASA-TLX was administered to measure cognitive load subjectively. The paired-samples *t*-test results revealed significant differences across several aspects of cognitive load. Mental demand was significantly higher for the MCT (*M* = 13.56, *SD* = 4.27) compared to the VST (*M* = 7.88, *SD* = 4.60), (t(32) = 5.20, *p* < 0.001). Participants rated their performance as significantly lower on the MCT (*M* = 10.91, *SD* = 5.24) than on the VST (*M* = 14.48, *SD* = 3.79, (t(32) = -3.17, *p* = 0.02). The perceived effort by participants was significantly greater for the MCT (*M* = 13.34, *SD* = 3.73) than for VST (*M* = 10.26, *SD* = 4.67, (t(32) = 5.20, *p* = 0.005). Frustration levels were also much higher for the MCT (*M* = 11.32, *SD* = 4.84) compared to the VST (*M* = 7.05, *SD* = 5.35, (t(32) = 5.20, *p* < 0.001). However, physical and temporal demand did not show a significant difference between the two tasks (*p* > 0.05). The DSPAN task, assessing the working memory, was performed once before the main tasks. The paired-samples *t*-test result showed a significant difference between the forward task (*M* = 6.62, *SD* = 1.21), and the backward task (*M* = 7.24, *SD* = 1.02), (t(32) = -2.25, *p* = 0.03).

### Linear discriminant analysis

In the experimental procedure, the intrinsic and extraneous cognitive load was induced with four levels of difficulty by the mental calculation and visual search tasks respectively. The descriptive statistics for each level of difficulty in both tasks are presented in Table 1. Linear discriminant analysis (LDA) was performed to differentiate between the low and high levels of CL in both task types, the mental calculation (short vs long calculations) and visual search (parallel vs serial search), and to differentiate at the same time between task types.

Before running the LDA, we report reliability scores for the predictors. The reliability of the physiological data was evaluated using split-half reliability estimated for 5000 random splits,^74^ considering the difficulty levels of the tasks. The results are presented for the highest and lowest Spearman-Brown coefficients. The reliability for fixation duration was between 0.74 and 0.87, for the low ICL and high ICL trials in the mental calculation task, respectively. For saccade amplitude, the high ICL trials of MCT had the lowest coefficient of 0.49, whereas the low ECL trials of VST showed 0.80. For the $\mathscr {K}$ coefficient, the high ECL trials in the visual search task had 0.84, and the low ICL trials of the mental calculation task showed a coefficient of 0.58. For heart rate variability, the lowest reliability belonged to the low ICL trials of the mental calculation task with a coefficient of 0.05, while the low ECL trials of the visual search task had 0.87. For the Low-High Index of Pupillary Activity, the low ICL trials of the mental calculation task indicated 0.70, and the low ECL trials of the visual search task had a coefficient of 0.82. For pupil dilation, the high ECL trials of the visual search task had the lowest coefficient of 0.66, while the low ICL and high ICL trials of the mental calculation task indicated a coefficient of 0.99.

#### Preliminary LDA models

The analyses start by running two separate LDA simpler models. The first model was to differentiate between the CL types (intrinsic vs. extraneous) and the second model focused on the separation between CL levels (low vs. high).

The first model separating intrinsic and extraneous cognitive load had a high mean classification accuracy of 0.986. The model produced one function with the highest coefficients for $\mathscr {K}$ coefficient ($\beta \!=\!-18.29$), average fixation duration ($\beta \!=\!13.28$), and saccade amplitude ($\beta \!=\!-11.09$). HRV ($\beta \!=\!0.17$) and LHIPA ($\beta \!=\!-0.15$) also contributed to this differentiation. The second model distinguishing levels of cognitive load (low vs. high) regardless of the task type achieved an average accuracy of 0.671. The model produced one discriminatory function with the highest coefficients for $\mathscr {K}$ coefficient ($\beta \!=\!-1.53$), average fixation duration ($\beta \!=\!1.46$), LHIPA ($\beta \!=\!0.73$), and saccadic amplitude ($\beta \!=\!-0.62$).

#### Discrimination of type and level of cognitive load

The main LDA model, discriminating between four groups, achieved an overall classification accuracy of $0.6538 ~(95\% ~\textit{CI:}~ 0.5956 - 0.7089), ~\textit{p} < 0.001, ~\textit{Kappa} = 0.537$, with the following prior probabilities (proportion of each group in the training data) $27.17\%$ for low ICL trials in the mental calculation task, $27.34\%$ for high ICL trials in the mental calculation task, $22.83\%$ for low ECL trials in the visual search task, and $22.65\%$ for high ECL trials in the visual search task. The balanced accuracy, sensitivity, and specificity of predictions in each group were as follows (0.752,  0.653,  0.851) for low ICL trials in the mental calculation task, (0.728,  0.589,  0.865) for *high ICL* trials in the mental calculation task, (0.788,  0.589,  0.914) for low ECL trials in the visual search task, and (0.814,  0.723,  0.905) for high ECL trials in the visual search task. Precision, recall, and F1 score calculated to evaluate the performance of the classification model in each group were as follows (0.622, 0.654, 0.638) for low ICL trials in the mental calculation task, (0.622, 0.590, 0.605) for high ICL trials in the mental calculation task, (0.694, 0.662, 0.677) for low ECL trials in the visual search task, and (0.691, 0.723, 0.707) for high ECL trials in the visual search task.

**Fig. 3:**
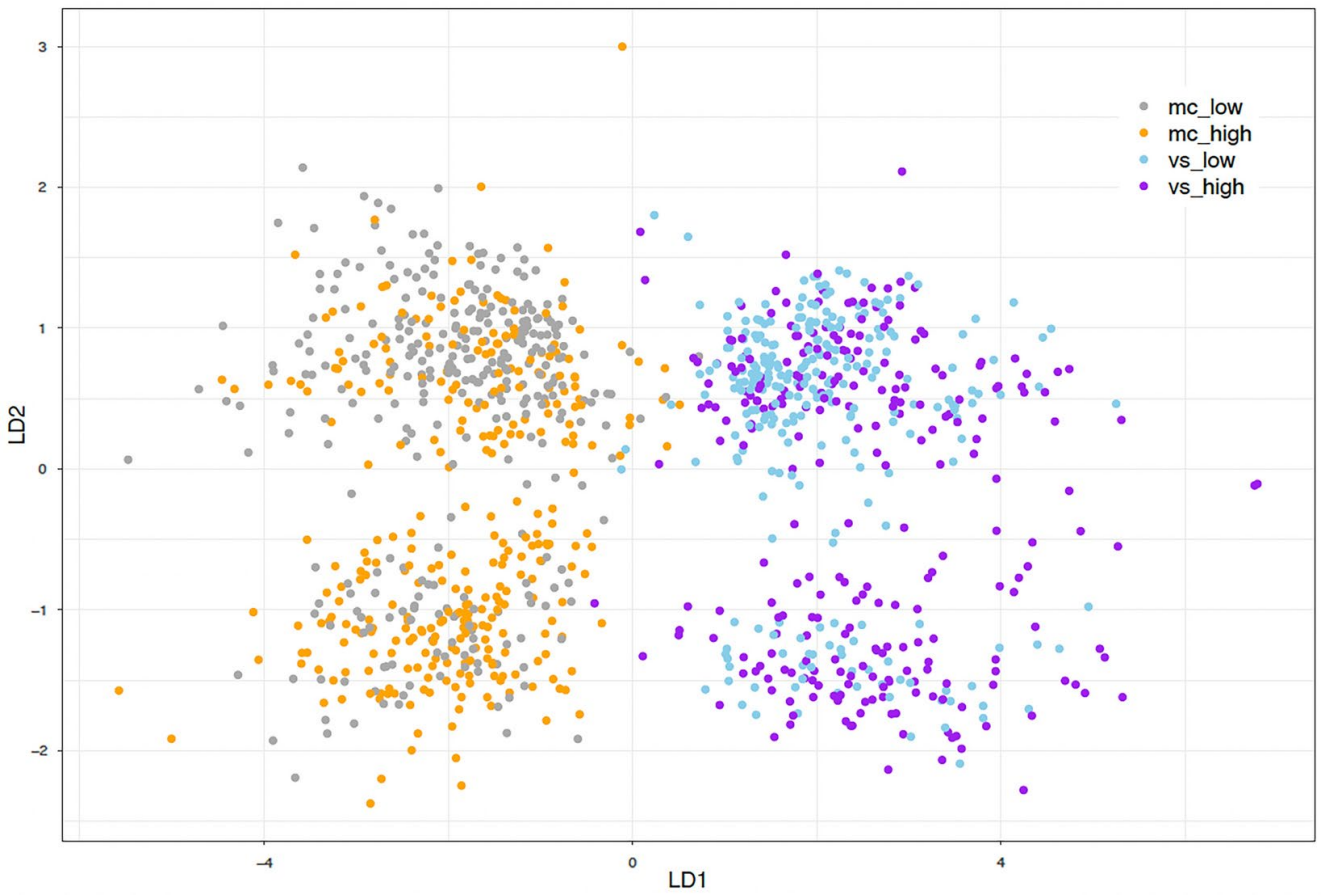
Classification results on cognitive load type and difficulty level.

**Fig. 4:**
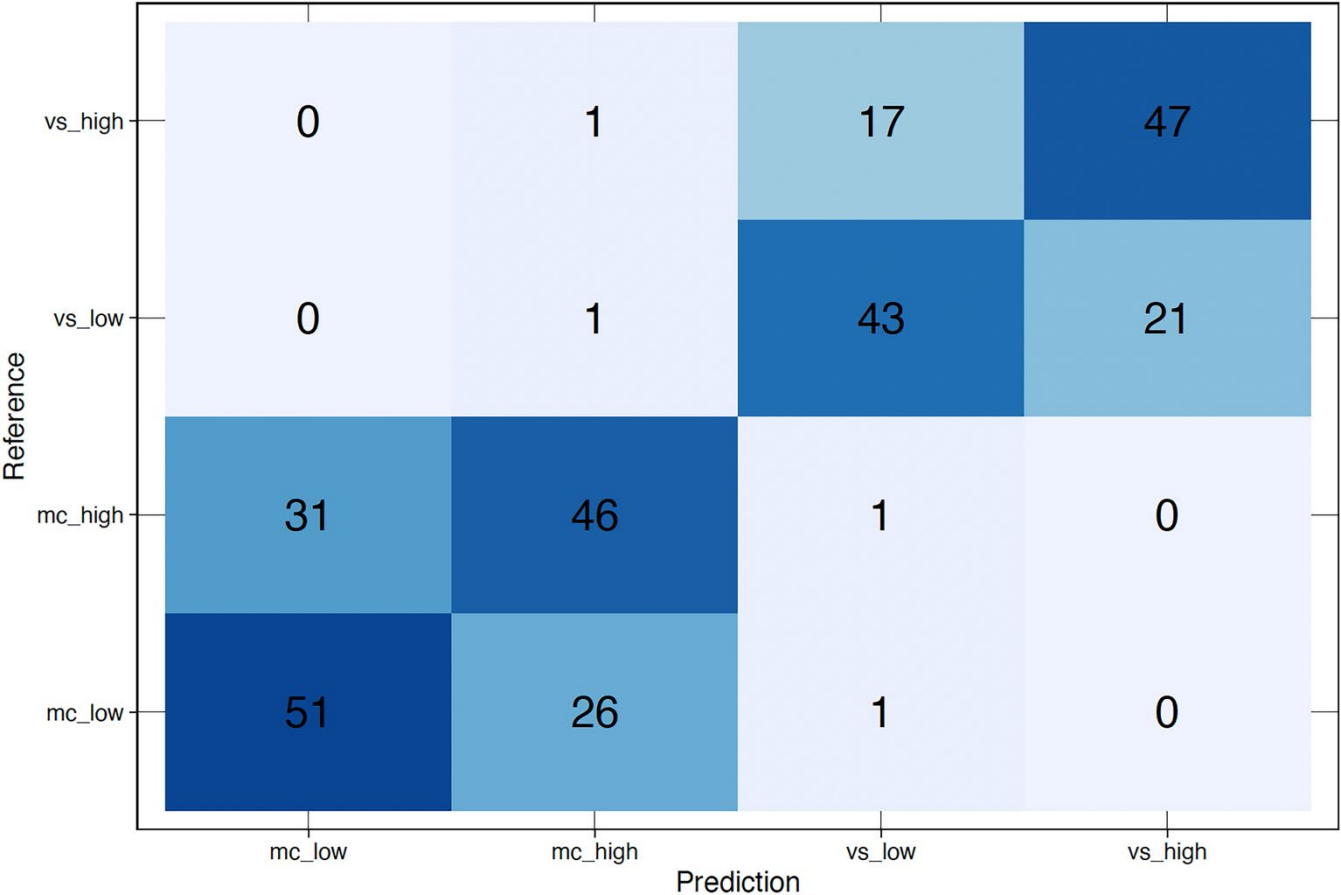
Confusion Matrix of the LDA classification model.

**Table 2:** Coefficients of Linear Discriminant.

Predictor	LD1	LD2	LD3
Fixation duration	13.428	-0.197	0.999
Saccade amplitude	-11.100	-0.363	-0.879
$\mathscr {K}$ coefficient	-18.297	-0.048	0.560
HRV	-0.195	0.140	0.516
GSR	0.065	0.104	-0.065
LHIPA	-0.138	-0.744	0.218
Pupil dilation	-0.046	-0.227	-0.112
DSPAN	-0.029	-0.076	-0.161

The LDA results reveal distinct linear combinations of the predictor variables that effectively separate the four groups. The LDA model revealed three discriminatory functions (see Table 2 for linear discriminant coefficients of each function). The following proportions of the trace for each linear discriminant indicate the amount of variance explained by each function, $97.25\%$ for LD1, $1.98\%$ for LD2, and $0.77\%$ for LD3. The classification results visualisation on X-Y dimensions representing two functions with the highest power (Figure 3) show a relatively clear classification of all cases into the four groups distinguished by task type and trial difficulty. Figure 3 and confusion matrix, a performance metric of discriminant analysis (see Figure 4), suggest that the LD1 function separates successfully between task types (mental calculation and visual search). The first linear discriminant function (LD1) is also the most powerful, accounting for over $97\%$ of the variance, primarily influenced by predictors such as *average fixation duration*, *saccade amplitude*, and $\mathscr {K}$ *coefficient*. However, looking at the group means of standardized predictors (Table 3) suggests that also *pupil dilation* might be considered a valued predictor of task type.

**Table 3:** Means for standardized predictors within each group: low CL trials in the mental calculation task (*mc_low*), high CL trials in the mental calculation task (*mc_high*), low CL trials in the visual search task (*vs_low*), high CL trials in the visual search task (*vs_high*).

Group	Fixation Duration	Saccade Amplitude	$\mathscr {K}$	HRV	GSR	LHIPA	Pupil Dilation	DSPAN
*mc_low*	-0.202	-0.263	0.113	0.488	-0.095	-0.248	0.214	0.035
*mc_high*	-0.106	0.261	-0.140	0.180	-0.100	0.302	0.319	-0.035
*vs_low*	-0.082	0.002	-0.167	-0.414	0.136	-0.278	-0.340	-0.014
*vs_high*	0.453	-0.001	0.201	-0.386	0.099	0.213	-0.300	0.014

The second and third discriminant functions (LD2 and LD3) contribute much less to the overall discrimination but still provide additional nuances in the separation of the groups especially between high and low CL trials of both experimental tasks (LD2), with *LHIPA* being notable in LD2 and *HRV* in LD3. The group means of standardized predictors (Table 3) indicate that *HRV* is sensitive in response to high and low ICL trials in mental calculation task. Surprisingly, *GSR* contribution to each discrimination function is low although, again group means (Table 3) might suggest its mild sensitivity in response to cognitive load type.

**Fig. 5:**
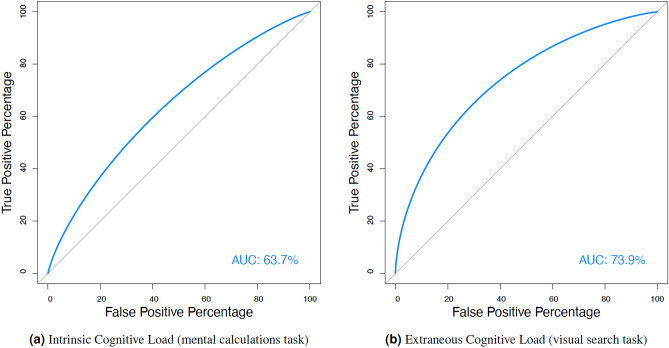
Receiver operating characteristic (ROC) curves with AUC values of distinctions between low and high cognitive load in intrinsic cognitive load task (5a) and extraneous cognitive load task (5b).

Finally, the ROC curves analysis, a measure of the model’s discriminatory ability, was performed separately for comparisons between low and high CL trials in each task type, the mental calculation (Figure 5a) and the visual search (Figure 5b). It shows that the area under the curve (AUC) ranges between $63.73\%$ and $73.94\%$ which indicates a meaningful level^75^ of discrimination quality between low and high cognitive load in both tasks based on the linear combination of discrimination functions with first-order and second-order eye-movement metrics and biometric predictors.

## Discussion

The main objective of this study was to examine predictive power for cognitive load types (intrinsic vs. extraneous) and levels (low vs. high) using eye movements (fixation characteristics, ambient-focal attention dynamics, and pupil-related measures) and other physiological metrics (HRV and GSR) with a predictive machine learning model. The applied Linear Discriminant Analysis model yielded the anticipated outcomes, demonstrating satisfactory discriminatory power in discerning high and low levels of induced cognitive load of different types.

The significance of the results lies in the capability of a single LDA model to effectively discern between different types and levels of cognitive load. First, we observed that fixation duration differentiated difficulty levels in the visual search task (extraneous cognitive load). Saccade amplitude differentiated difficulty levels in mental calculation task (intrinsic cognitive load). However, the $\mathscr {K}$ coefficient distinguished difficulty levels in both cognitive load types. Second, pupil dilation (the percentage change in pupil size in relation to baseline) differentiates between types of cognitive load, whereas LHIPA contributes to the understanding of the cognitive load levels, regardless of the load type. Finally, the contribution of bio-metrics, HRV and GSR, to the discriminatory functions is relatively low. Both measures tend to differentiate between cognitive load types, with HRV being also responsive to task difficulty, especially in the context of intrinsic cognitive load reflected in mental calculations.

Physiological measurements provide a continuous and real-time evaluation for the assessment of cognitive load in a variety of human-computer interaction studies^76, 77^. However, previous studies have also demonstrated that the reliability of continuous data can be affected by fluctuations during active task performance due to changes in participant engagement^78, 79^ and extraneous factors, such as sample size, eye-tracker resolution, and task duration^80–82^. For example, HRV reliability is influenced by the duration of the measurement period^82^. Chapman et al.^83^ investigated the impact of task duration, comparing long and short durations (one versus five minutes). The authors observed slight differences between the short and long durations while measuring HRV. Also, Ruangsuphaphichat et al.^84^ demonstrated that that short-term assessments (between five and 10 minutes) result in fluctuations between moderate and excellent reliability. In our study, brief duration of measurements (the answer screen in MCT and the stimulus screen for the VST) might have resulted in varied reliability scores for different experimental conditions. We postulate to prolong the trial durations in future studies to increase HRV reliability values.

Recent empirical inquiries into the relationship between eye-movement metrics and cognitive load have primarily focused on first-order metrics (e.g. fixation duration and pupil dilation) and intrinsic load. However, the results from these investigations have been inconclusive. While DeRivercourt et al.^85^ and Mallick et al.^22^ reported a reduction in fixation duration with increased intrinsic cognitive load, more recent literature has indicated an opposite relationship^28, 29^. Our findings support the current literature, demonstrating that fixation duration increases with heightened task demands, presumably indicating higher intrinsic cognitive load. In the realm of intrinsic cognitive load assessment, pupil dilation stands out as a critical metric^36^. Research has consistently demonstrated the reliability of pupil diameter as a measure of intrinsic cognitive load. Hess and Polt’s foundational work^34^ first associated an increase in pupil diameter with higher cognitive load. Kahnemann and Beatty^86^ reported an increase in pupil dilation with task difficulty^86^. More recent studies, such as that by Gavas et al.^87^ have shown that percentage change in pupil dilation (PCPD) can effectively discern between low and high general cognitive load during mental tasks. Also, Zu et al.^4^ reported that pupil diameter changes have been larger with increasing cognitive load in a multimedia learning setting. Aligning with this line of inquiry, our findings support the notion that pupil dilation distinguishes between ease and difficulty in tasks inducing not only intrinsic but also extraneous loads alike (see also Duchowski et al.^33^, Krejtz et al.^38, 60^).

It should be noted that pupil dilation can be affected by exposure to a new stimulus, such as mental effort or luminance, at the same time. Consequently, some studies have employed baseline correction techniques (the difference between measures taken during the baseline screen and tasks) to enhance the statistical power regarding fluctuations in pupil size^88^. This calculation, based on pre-task pupil size, may provide a reliable basis for interpreting the impact of mental tasks on pupil dilation^89^. Furthermore, Peysakhovich et al.^90^ conducted a study in which the task-evoked pupil response was found to depend on different luminance conditions. The authors reported that task-evoked and luminance factors were expressed differently depending on the frequency. While the ratio between low and high frequency (LF/HF ratio) of the pupillary signal is sensitive to tasks requiring mental effort, it is not sensitive to luminance^90^. For this reason we also measured LHIPA, which reflects the pupillary activity as a ratio between low/high frequency^33^. In the present study, the luminosity of the screens was maintained at a constant level throughout the tasks. It was therefore assumed that pupil dilation reflected task-related changes based on cognitive load.

The use of biometrics like heart rate variability (HRV) and skin conductance for discerning cognitive load has been also investigated previously. Our results demonstrated a negative relationship between HRV and increased cognitive load, consistent with prior research^8, 51^. However, a separate study reported a positive association between intrinsic cognitive load and HRV^52^. The observed negative relationship suggests that as the cognitive demands of the mental calculation task increase, the autonomic nervous system responds with reduced HRV, reflecting higher levels of stress, arousal, or cognitive load. Prior studies suggested an increase in GSR and a decrease in HRV with rising task demand for cognitive load^51, 55^.

The novelty of the present findings is also attributed to the utilization of second-order eye movement metrics, particularly the Low-High Index of Pupillary Activity (LHIPA) and the $\mathscr {K}$ coefficient of ambient-focal attention, for the purpose of cognitive load prediction. Previous research has established LHIPA’s sensitivity to task load^40, 60^. However, this study is the first to utilize it as an input variable in a predictive model. In line with Duchowski et al.’s^40^ decrease in LHIPA associated with increased difficulty, this model exhibited a different way of discriminating task difficulty. This result may stem from variations in experimental design or the task nature.

The $\mathscr {K}$ coefficient has been validated as a robust measure of the attentive momentary state, encompassing both ambient and focal modes. Focal mode of attention is associated with the higher demand of cognitive resources for information processing^25, 26, 91^. As expected, the mean value of $\mathscr {K}$ for the serial search, the challenging conditions in the task, showed greater focal visual attention ($\mathscr {K} > 0$). Additionally, the $\mathscr {K}$ coefficient demonstrated significant efficacy in distinguishing between load types, likely due to the inherent differences in the nature of the experimental tasks (visual search versus mental calculation). This interpretation however might not hold when considering similarities in the visual design of both tasks.

To achieve a sustainable measurement of cognitive load, it is necessary to employ a single-factor manipulation within the context of a controlled experimental setting^92^. In the present study, it was assumed that the difficulty levels of the calculations affected mainly the intrinsic cognitive load, whereas the visual design of the visual search task mainly contributed to the extraneous cognitive load. Distracting details during visual processing lead to high extraneous load^92, 93^. For example, similar to our visual search task, in a series of experiments conducted by Liu et al.^94^, Gabor patches were used to investigate the impact of cognitive load on visual processing. Park et al.^95^ conducted an eye-tracking study to investigate the effect of distracting details on cognitive load. Their results indicated that learning processing, as measured by eye metrics, was low during conditions with distracting details. Finally, in a review of studies including distracting details, Rey^96^ reported that the majority of studies yielded large effect sizes (up to 2.29 Cohen’s *d*) supporting the effect of distracting details on increasing working memory and extraneous cognitive load.

According to Cognitive Load Theory,^1^ the inherent complexity of the material itself is related to intrinsic cognitive load, and the exposure of irrelevant elements is related to extraneous cognitive load. The mental calculations task (MCT) in this study forced participants to integrate information into their existing knowledge structure. Therefore, in MCT, intrinsic cognitive load increases with the complexity of the calculations, rather than with their visual design or redundant factors^4, 5^. The visual search task (VST), however, requires participants to focus on finding a target among distracting elements. The effort induced by irrelevant visual information, (e.g., focusing on finding the target among randomly oriented Gabor patches) consumes cognitive resources and leads to changes in extraneous load^97, 98^. Thus, in VST, the extraneous cognitive load changes due to manipulation of task design^4, 5^. Highlighting the differences between these two tasks based on CLT is crucial because the type of cognitive load directly affects how learners use their cognitive resources. Understanding how tasks assumed to trigger different types of cognitive load affect working memory and learning may be beneficial for further studies in educational scenarios. On the one hand, if the design and organization of the learning material exceed the capacity of working memory, it will be challenging to engage in cognitive processing^99^. On the other hand, measuring cognitive load is not a straightforward process in the absence of an educational context or learning objectives. However, tasks with distracting details (e.g., the hit condition in visual search task) represent a possible approach for investigating working memory capacity.

Although the present study has met our expectations, we also acknowledge that it is not free from limitations warranting further investigation. First, the influence of fatigue as a secondary factor on cognitive functioning and the potential increase in cognitive load due to the split attention effect needs consideration^100^. Second, the relatively small sample size may limit result generalizability, as Linear Discriminant Analysis is sensitive to small sample sizes, potentially leading to overfitting and reduced robustness and efficiency^101, 102^. Third, the duration of the measurement period for the task may be considered a potential limitation in terms of achieving a high level of reliability in the physiological measurements. Finally, future studies may use a different manipulation of task difficulty with more trials to boost the discrimination of task levels, consequently increasing the model’s classification accuracy.

In conclusion, the study presents a predictive model for intrinsic and extraneous cognitive load and task levels using oculo- and biometric indicators. To our knowledge, no prior research has explored the use of various physiological metrics to predict both cognitive load types and their levels within a machine-learning framework. Furthermore, the model incorporates second-order metrics of eye movements as a novel approach to enhance classification capability.

## Data Availability

The datasets generated during the current study are available in the Zenodo repository, accessible via DOI: 10.5281/zenodo.14173461.
